# Analysis of risk factors for ureteral stricture and tumor recurrence after transurethral resection of bladder tumor in paraureteral bladder cancer

**DOI:** 10.3389/fsurg.2025.1680837

**Published:** 2025-10-15

**Authors:** Rui Meng, Yu Han, Zhipeng Zhai

**Affiliations:** Department of Urology, YuQuan Hospital, Tsinghua University, Beijing, China

**Keywords:** TURBT, paraureteral bladder cancer, tumor recurrence, ureteral stricture, diabetes mellitus

## Abstract

**Background:**

This study explores the risk factors for ureteral stricture and tumor recurrence after transurethral resection of bladder tumor (TURBT) in patients with paraureteral bladder cancer, aiming to provide references for clinicians in their treatment practices.

**Methods:**

A retrospective analysis was conducted on 164 patients with paraureteral bladder cancer who underwent TURBT from April 2017 to July 2024, among whom 133 patients had complete clinical data and follow-up data. Univariate and multivariate logistic regression analyses were used to identify the risk factors for ureteral stricture and tumor recurrence after TURBT in paraureteral bladder cancer.

**Results:**

The incidence of ureteral stricture after TURBT in patients with paraureteral bladder cancer was 11.28% (15/133), and the recurrence rate of bladder cancer within 1 year after surgery was 21.80% (29/133). Multivariate logistic regression analysis showed that diabetes mellitus (*p* = 0.021) and tumor diameter (*p* = 0.002) were independent risk factors for ureteral stricture within 1 year after TURBT. Additionally, multivariate logistic regression analysis revealed that sex (*p* = 0.021), diabetes mellitus (*p* = 0.008), and pathological T stage (*p* = 0.002) were independent risk factors for bladder tumor recurrence within 1 year after TURBT.

**Conclusion:**

Diabetes mellitus and tumor diameter are independent risk factors for ureteral stricture within 1 year after TURBT in patients with paraureteral bladder cancer. Sex, diabetes mellitus, and pathological T stage are independent risk factors for bladder tumor recurrence within 1 year after TURBT in these patients.

## 1 Introduction

Bladder cancer is one of the most common malignancies in the genitourinary system, categorized into muscle-invasive and non-muscle-invasive bladder cancer (NMIBC). NMIBC accounts for 70%–75% of all bladder cancer cases (1–3). Transurethral resection of bladder cancer (TURBT) is the primary treatment for NMIBC; however, the risk of tumor recurrence postoperatively remains as high as 45% (4, 5).

Bladder cancer located in different anatomical sites may exert varying impacts on post-TURBT recurrence and survival outcomes (6–10). Among the nine bladder locations—anterior wall, bladder neck, dome, lateral wall, posterior wall, trigone, urachus, ureteric orifice, and overlapping lesions—current research primarily focuses on the bladder neck, with relatively fewer studies on paraureteral bladder cancer. In fact, bladder cancer around the ureteral orifice also deserves our attention. The anatomical structure of this area is delicate, and TURBT procedures are prone to causing ureteral stricture, while also increasing the risk of postoperative recurrence.

This study aims to analyze the risk factors for ureteral stricture and tumor recurrence after TURBT in paraureteral bladder cancer, providing clinical references for the management of such cases.

## 2 Methods

**Figure 1:**
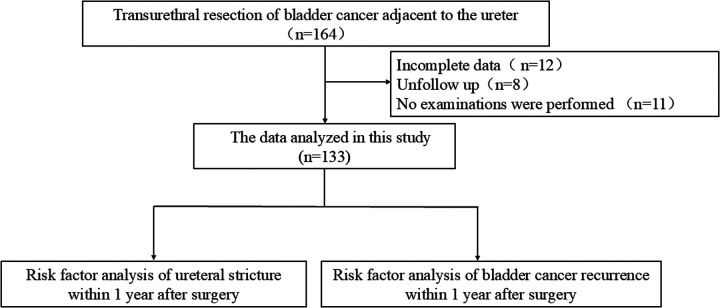
The study flow chart.

A total of 164 patients with paraureteral bladder cancer who underwent TURBT at the Department of Urology, Miyun Hospital of Peking University First Hospital and Cangzhou Central Hospital between April 2017 and July 2024 were included. Among them, 133 patients had complete baseline and follow-up data (Figure 1). The cohort included 38 cases of solitary bladder cancer and 74 cases of multiple bladder cancer (defined as two or more bladder tumors). Clinical data and follow-up data were collected, including sex, age, body mass index (BMI), smoking status, hypertension, diabetes mellitus, pathological stage, pathological grade, number of tumors, and tumor diameter. Postoperative follow-up assessed tumor recurrence and ureteral stricture within 1 year after TURBT. The operation was performed by two chief surgeons, both of whom have more than 10 years of experience in urological surgeries and extensive experience in transurethral resection of the bladder.

Parauterine bladder cancer was defined as a tumor appearing at a maximum distance of 1 cm from the edge of the ureteric orifice (11). The distance was measured using the diameter of the resection loop, which corresponds to 7 mm. Definition of ureteral stricture: Ureteral stricture is defined as a condition where computed tomography urography (CTU) shows the diameter of the ureteral lumen is less than 2 mm, or endoscopic examination reveals narrowing or even occlusion of the ureteral lumen. Diagnostic criteria for bladder cancer recurrence: The diagnosis of bladder cancer recurrence is confirmed when abnormal lesions are detected by cystoscopy and pathologically confirmed as urothelial carcinoma through biopsy.

Inclusion criteria: a. patients with NMIBC; b. First-time TURBT; c. Tumor located only around the ureteral orifice without involving the ureter; d. Pathological stage Ta or T1; e. Complete clinical and follow-up data. Exclusion criteria: a. History of other malignancies; b. Coagulopathy; c. Cardiopulmonary dysfunction.

This study was conducted in accordance with the principles of the Declaration of Helsinki (revised 2013) and approved by the Ethics Committee of Peking University First Hospital-Miyun Hospital. Informed consent was waived for the retrospective analysis.

### 2.1 Surgical procedure

After successful anesthesia, the patient was placed in the lithotomy position, with limbs properly fixed and protected to ensure adequate exposure of the surgical field. The perineal area and surrounding regions were disinfected with iodine, and a sterile drape was applied to establish the surgical field. A 24F 12° resectoscope (Hawk, Hangzhou, China) was inserted into the bladder via the urethra. The bladder was distended and irrigated with 5% mannitol to assess bladder status and confirm tumor location and quantity. Resection was performed using the cutting mode (80–100W) within a 1 cm margin around the tumor. Tumor tissues were completely evacuated using negative pressure, and all resected fragments were collected in sterile specimen containers, labeled, and sent for pathological examination immediately. The resectoscope was then switched to coagulation mode (60–80W) for precise hemostasis of visible bleeding points. Small blood vessels were treated with spot coagulation to avoid excessive bladder mucosa damage from extensive coagulation. After hemostasis, the bladder was re-distended with mannitol to carefully inspect the surgical site and all bladder walls, ensuring no active bleeding, residual tumor tissue, or bladder perforation. The irrigation was stopped, and the resectoscope was withdrawn slowly and gently, with careful observation for urethral mucosa injury. A 3-way large balloon catheter (Bard, New Jersey, USA) was inserted for bladder irrigation.

### 2.2 Statistical analysis

Statistical analyses were performed using SPSS 22.0 (IBM Corp., Armonk, NY, USA). Normally distributed continuous data were expressed as mean ± standard deviation, while skewed data were described as median (range). For continuous variables, *t*-tests were used for normally distributed data, and Mann–Whitney *U*-tests for non-normally distributed data. Categorical variables were analyzed using the *χ*^2^ test or Fisher's exact test. Variables significantly associated with “ureteral stricture occurrence” and “bladder cancer recurrence” (*P* < 0.05) were first screened out using univariate logistic analysis. Subsequently, these variables with statistical significance were included in the multivariate logistic analysis. A *p*-value < 0.05 was considered statistically significant.

## 3 Results

### 3.1 Baseline characteristics

Baseline data of patients are shown in Table 1. A total of 133 patients were included, with a mean age of 66.98 ± 10.36 years and a mean tumor diameter of 2.86 ± 1.53 cm. The incidence of ureteral stricture after TURBT was 11.28% (15/133), and the 1-year postoperative recurrence rate of bladder cancer was 21.80% (29/133). No deaths occurred within 1 year postoperatively.

**Table 1:** Basic characteristics of the patients.

Variable	Mean (SD) or n/N
Patients	133
Mean age (years)	66.98 ± 10.36
BMI (kg/m^2^)	24.76 ± 2.95
Sex, *n* (%)	
Male	112(84.21)
Female	21(15.79)
Hypertension, *n* (%)	
Yes	50(37.59)
No	83(62.41)
Diabetes mellitus, *n* (%)	
Yes	13(9.77)
No	120(90.23)
Smoking, *n* (%)	
Yes	41(30.83)
No	92(69.17)
Number of tumors, *n* (%)	
Single	44(33.08)
Multiple	89(66.92)
Pathological T, *n* (%)	
Ta	52(39.10)
T1	81(60.90)
Histological grade, *n* (%)	
Low	49(36.84)
High	84(63.16)
Tumor diameter(cm)	2.86 ± 1.53
Postoperative ureteral stricture, *n* (%)	
Yes	15(11.28)
No	118(88.72)
Postoperative tumor recurrence, *n* (%)	
Yes	29(21.80)
No	104(79.20)

BMI, body mass index.

### 3.2 Risk factors for ureteral stricture after TURBT

Clinical data of patient's ureteral stricture group and non-ureteral stricture after TURBT are shown in Table 2. The proportion of diabetic patients in the stricture group was 26.67% (4/15), significantly higher than that in the non- ureteral stricture group (7.63%, 9/118; *p* = 0.021). The mean tumor diameter in the stricture group was 4.07 ± 0.97 cm, significantly larger than that in the non- ureteral stricture group (2.71 ± 1.51 cm; *p* = 0.001).

**Table 2:** Comparison between the ureteral stricture group and the non-ureteral stricture group after TURBT.

Variable	Ureteral stricture group	Non-ureteral stricture group	*p*-value
Patients	15	118	
Mean age (years)	63.13 ± 12.00	67.47 ± 9.97	0.131
BMI (kg/m^2^)	24.94 ± 2.74	24.74 ± 2.96	0.813
Sex, *n* (%)			0.782
Male	13(86.67)	99(83.90)	
Female	2(13.33)	19(16.10)	
Hypertension, *n* (%)			0.199
Yes	8(53.33)	42(35.59)	
No	7(46.67)	76(64.41)	
Diabetes mellitus, *n* (%)			0.021
Yes	4(26.67)	9(7.63)	
No	11(73.33)	109(92.37)	
Smoking, *n* (%)			0.440
Yes	6(40.00)	35(29.66)	
No	9(60.00)	83(70.34)	
Number of tumors, *n* (%)			0.575
Single	4(26.67)	40(33.90)	
Multiple	11(73.33)	78(66.10)	
Pathological T, *n* (%)			0.524
Ta	7(46.67)	45(38.14)	
T1	8(53.33)	73(61.86)	
Histological grade, *n* (%)			0.765
Low	5(33.33)	44(37.29)	
High	10(66.67)	74(62.71)	
Tumor diameter(cm)	4.07 ± 0.97	2.71 ± 1.51	0.001

Univariate and multivariate logistic regression analyses identified diabetes mellitus (*p* = 0.021) and tumor diameter (*p* = 0.002) as independent risk factors for ureteral stricture within 1 year after TURBT (Table 3).

**Table 3:** Univariate and multivariate logistic regression analysis of ureteral stricture after TURBT for parauterine bladder cancer.

Characteristic	Univariate analysis		Multivariate analysis	
	OR (95% CI)	*P*-value	OR (95% CI)	*P*-value
Mean age	0.960(0.909–1.013)	0.134		
BMI	1.022(0.852–1.227)	0.811		
Sex	1.247(0.260–5.981)	0.782		
Hypertension	0.497(0.168–1.466)	0.205		
Diabetes mellitus	0.231(0.061–0.876)	0.031	0.183 (0.043–0.775)	0.021
Smoking	0.648(0.214–1.960)	0.442		
Number of tumors	0.709(0.212–2.369)	0.576		
Pathological T	0.705(0.239–2.075)	0.525		
Histological grade	0.841(0.270–2.620)	0.765		
Tumor diameter	1.725(1.212–2.457)	0.002	1.783 (1.232–2.579)	0.002

### 3.3 Risk factors for tumor recurrence after TURBT

Clinical data of patient's tumor recurrence group and non-tumor recurrence group after TURBT are shown in Table 4. The proportion of male patients in the recurrence group was 68.97% (20/29), significantly lower than that in the non- tumor recurrence group (88.46%, 92/104; *p* = 0.011). The proportion of diabetic patients in the recurrence group was 24.14% (7/29), significantly higher than that in the non- tumor recurrence group (5.77%, 6/104; *p* = 0.001). In the recurrence group, 20.69% (6/29) of tumors were pathologically staged as Ta, significantly lower than the 44.23% (46/104) in the non- tumor recurrence group (*p* = 0.022).

**Table 4:** Comparison between the tumor recurrence group and the non-tumor recurrence group after TURBT.

Variable	Tumor recurrence group	Non-tumor recurrence group	*p*-value
Patients	29	104	
Mean age (years)	69.90 ± 8.32	66.16 ± 10.69	0.089
BMI (kg/m^2^)	25.04 ± 3.24	24.69 ± 2.84	0.573
Sex, *n* (%)			0.011
Male	20(68.97)	92(88.46)	
Female	9(31.03)	12(11.54)	
Hypertension, *n* (%)			0.089
Yes	15(51.72)	35(33.65)	
No	14(48.28)	69(66.35)	
Diabetes mellitus, *n* (%)			0.004
Yes	7(24.14)	6(5.77)	
No	22(75.86)	98(94.23)	
Smoking, *n* (%)			0.625
Yes	8(27.59)	33(31.73)	
No	21(72.41)	71(68.27)	
Number of tumors, *n* (%)			0.530
Single	11(37.93)	33(31.73)	
Multiple	18(62.07)	71(68.27)	
Pathological T, *n* (%)			0.022
Ta	6(20.69)	46(44.23)	
T1	23(79.31)	58(55.77)	
Histological grade, *n* (%)			0.567
Low	12(41.38)	37(35.58)	
High	17(58.62)	67(64.42)	
Tumor diameter(cm)	3.10 ± 1.18	2.80 ± 1.59	0.341

Univariate and multivariate logistic regression analyses identified sex (*p* = 0.021), diabetes mellitus (*p* = 0.008), and pathological T stage (*p* = 0.002) as independent risk factors for tumor recurrence within 1 year after TURBT (Table 5).

**Table 5:** Univariate and multivariate logistic regression analysis of bladder cancer recurrence after TURBT for parauterine bladder cancer.

Characteristic	Univariate analysis		Multivariate analysis	
	OR (95% CI)	*P*-value	OR (95% CI)	*P*-value
Mean age	1.036(0.994–1.079)	0.091		
BMI	1.041(0.905–1.198)	0.570		
Sex	0.290(0.108–0.780)	0.014	0.326 (0.114–0.934)	0.037
Hypertension	2.051(0.890–4.728)	0.092		
Diabetes mellitus	5.091(1.557–16.647)	0.007	0.181 (0.051–0.645)	0.008
Smoking	0.797(0.319–1.987)	0.626		
Number of tumors	1.315(0.558–3.905)	0.531		
Pathological T	3.040(1.143–8.087)	0.026	3.202 (1.134–9.045)	0.028
Histological grade	1.278(0.551–2.964)	0.567		
Tumor diameter	1.154(0.950–1.403)	0.149		

## 4 Discussion

Bladder cancer is one of the most common malignancies in the urinary system, with a globally increasing incidence (12). Postoperative recurrence and complications in bladder cancer patients directly affect treatment efficacy, quality of life, and even survival, thus requiring close attention (13, 14). Differences in anatomical structure and physiological characteristics across bladder locations influence TURBT difficulty, completeness of tumor resection, and risk of postoperative complications. This study focuses on paraureteral bladder cancer, analyzing risk factors for post-TURBT ureteral stricture and tumor recurrence.

Liu et al. (15) reported that tumor location significantly impacts TURBT outcomes in NMIBC patients, with tumors in the anterior wall and bladder dome associated with poorer prognosis and higher recurrence risk. In fact, bladder cancer located at the bladder neck, bladder dome, and paraureteral region are clinical challenges (9, 16–18). The bladder neck is adjacent to the internal urethral orifice, with delicate surrounding tissues and limited space, making it prone to injury during surgery and incomplete tumor resection, thereby increasing recurrence risk. Tumors at the bladder dome are often concealed, leading to potential residual disease and higher recurrence rates.

Paraureteral bladder cancer is prone to both recurrence and ureteral stricture postoperatively. TURBT in this area may directly damage the ureteral orifice mucosa or surrounding tissues, triggering inflammation and scarring that result in ureteral stricture (19). Additionally, tumors in this location often adhere closely to the ureteral wall, with potential mucosal spread or ureteral invasion. Even after visible tumor resection, residual cells may recur locally. Ureteral stricture further impairs urine drainage, exacerbating bladder microenvironment abnormalities and indirectly promoting tumor recurrence. However, in reality, current research mainly focuses on subgroup analysis of bladder cancer located at the bladder neck and bladder dome, while studies on paraureteral bladder cancer are scarce. By analyzing the postoperative bladder cancer recurrence and ureteral stricture in 133 patients with paraureteral bladder cancer, this study fills the research gap in the subgroup of paraureteral bladder cancer. In particular, the risk factors for ureteral stricture after surgery for paraureteral bladder cancer have not been analyzed in any previous studies.

In this study, the incidence of ureteral stricture after TURBT was 11.28% (15/133), with diabetes mellitus (*p* = 0.021) and tumor diameter (*p* = 0.002) identified as independent risk factors for 1-year postoperative stricture. Roy Mano et al. (20) reported a 4% (3/79) incidence of significant ureterovesical junction stricture requiring endoscopic intervention in paraureteral bladder cancer patients after TURBT. The higher stricture rate in our study may be attributed to larger tumor diameters. Faba et al. (21) analyzed risk factors for ureteral stricture after TURBT in NMIBC involving the distal ureter, identifying tumor size ≥1.5 cm (HR 4.521, *p* = 0.023) and T1 stage (HR 8.525, *p* = 0.005) as risk factors, consistent with our finding that tumor diameter is an independent risk factor.

The impact of tumor diameter on ureteral stricture is directly related to the extent of anatomical invasion and the difficulty of surgical operation. In this study, the mean tumor diameter in the ureteral stricture group (4.07 ± 0.97 cm) was significantly larger than that in the non-stricture group (2.71 ± 1.51 cm, *P* = 0.001). The reason for this is as follows: when the tumor diameter exceeds 3 cm, its invasive range often approaches or even involves the “safe zone” within 5 mm around the ureteral orifice. During surgical resection, to ensure complete tumor removal (which requires a 1 cm margin from the tumor edge), the probability of damaging the mucosa and muscular layer of the ureteral orifice will inevitably increase. Meanwhile, the resection wound of a larger tumor is larger, and the energy output during electrocoagulation hemostasis may be conducted to the ureteral wall, causing local tissue thermal injury and significantly increasing the probability of postoperative scar tissue formation. This is consistent with the conclusion of Faba et al. (21) that “tumor size ≥1.5 cm is a risk factor for ureteral stricture after NMIBC surgery”.

For diabetic patients, the impact of hyperglycemia on postoperative ureteral stricture is reflected in two key links. On one hand, long-term hyperglycemia inhibits the proliferation of fibroblasts and the normal cross-linking of collagen, resulting in a decrease in the repair ability of the ureteral orifice and surrounding tissues, delayed healing of surgical wounds, and subsequent chronic inflammatory reactions (22). On the other hand, hyperglycemia reduces the phagocytic function of neutrophils and the activity of lymphocytes, increasing the risk of postoperative urinary tract infection. Infection further aggravates ureteral mucosal edema and submucosal fibrosis, eventually leading to lumen stricture (23). This echoes the conclusion of Roy Mano et al. (20) that “metabolic abnormalities may increase the risk of stricture after urinary system surgery”. However, this study is the first to clearly identify diabetes as an independent risk factor for stricture after paraureteral bladder cancer surgery, providing direct evidence for preoperative blood glucose management in such patients.

The 1-year postoperative recurrence rate in this study was 21.80% (29/133), with sex (*p* = 0.021), diabetes mellitus (*p* = 0.008), and pathological T stage (*p* = 0.002) as independent risk factors. Seung-Hwan Jeong et al. (6) identified multiple prior TURBTs, high tumor count, tumor location, tumor shape, incomplete resection, and high-grade tumors as recurrence risk factors. For paraureteral bladder cancer, Faba et al. (17) reported that T1 bladder lesions (HR 7.253, *p* = 0.001) and carcinoma *in situ* in the distal ureteral muscular layer (HR 6.850, *p* = 0.005) increase recurrence risk. T1 tumors infiltrate deeper into the lamina propria compared to Ta tumors, making complete resection more difficult and increasing residual tumor risk. T1 tumors also exhibit higher invasiveness, facilitating residual cell proliferation and recurrence (24).

Lu et al. (25) reported an association between diabetes mellitus and poor prognosis/recurrence in bladder cancer patients via meta-analysis but did not explore underlying mechanisms. We hypothesize that hyperglycemia contributes to recurrence by suppressing immune cell (e.g., lymphocyte, macrophage) activity, impairing tumor surveillance and clearance, and allowing residual tumor cells to proliferate.

For NMIBC, multiple studies indicate that women have a higher recurrence risk after TURBT and bacillus calmette-guérin therapy, attributed to differences in immunogenicity (26). However, we propose that anatomical differences may play a role: the smaller bladder capacity and shorter urethra in women reduce urinary drainage and flushing, facilitating residual tumor cell colonization and growth.

Notably, surgeon experience is a critical factor influencing post-TURBT recurrence and ureteral stricture. Ayman Kassem et al. (27) demonstrated that surgeon experience significantly impacts TURBT quality and NMIBC recurrence risk. In our study, all surgeons had over 10 years of experience, minimizing bias from varying expertise.

The risk factors for ureteral stricture and tumor recurrence after TURBT identified in studies also require our attention in clinical practice. For diabetic patients, we can conduct a comprehensive preoperative assessment of their blood glucose control and adjust hypoglycemic medications to reduce the impact of hyperglycemia on tissue repair and immune function. If the tumor is large, a ureteral stent can be placed after tumor resection to support the distal ureter and drain urine, thereby reducing postoperative edema and the occurrence of scar stricture. For patients with large tumors, preoperative imaging should be used to assess the anatomical relationship between the tumor and the ureteral orifice, and intraoperative damage to the ureteral orifice and surrounding tissues should be minimized. In addition to routine postoperative follow-up, urinary system computed tomography urography can be initiated earlier to detect early ureteral stricture and avoid delaying intervention. Based on the postoperative pathological T stage, more rigorous follow-up protocols should be adopted for patients with high T stages. For example, the interval between cystoscopies can be shortened to once every 2–3 months to dynamically monitor for signs of tumor recurrence and gain time for early intervention (such as secondary transurethral resection or radical surgery).

This study has certain limitations. First, the sample size of this study is small. Among the 133 samples, there are only 15 cases of ureteral stricture and 29 cases of tumor recurrence, which affects subgroup analysis and model stability, and may lead to false negatives. Second, this study is a retrospective study. Reliance on medical record data results in the lack of some information (such as smoking details and perfusion protocols), and there is selection bias, which limits the extrapolation of the results. Third, the sample size was not estimated in advance, so it is impossible to determine whether the current data are sufficient to detect the preset effect size, which reduces the reliability of the results. Future studies will adopt a prospective design, expand the sample size and improve data collection to enhance the credibility of the conclusions.

## Data Availability

The raw data supporting the conclusions of this article will be made available by the authors, without undue reservation.
